# Impact of MIF Gene Promoter Variations on Risk of Rheumatic Heart Disease and Its Age of Onset in Saudi Arabian Patients

**DOI:** 10.3389/fimmu.2016.00098

**Published:** 2016-03-14

**Authors:** Atiyeh M. Abdallah, Abdulhadi H. Al-Mazroea, Waleed N. Al-Harbi, Nabeeh A. Al-Harbi, Amr E. Eldardear, Yousef Almohammadi, Khalid M. Al-Harbi

**Affiliations:** ^1^West Midlands Regional Genetics Laboratory, Birmingham Women’s NHS Foundation Trust, Birmingham, UK; ^2^Pediatric Department, Maternity and Children Hospital, Ministry of Health, College of Medicine, Taibah University, Al-Madinah, Saudi Arabia; ^3^Security Forces Medical Centre, Al-Madinah, Saudi Arabia

**Keywords:** migration inhibitory factor, rheumatic heart disease, polymorphisms, age of onset, Saudi Arabia

## Abstract

Although macrophage migration inhibitory factor (MIF) has consistently been shown to be an important immune modulator, data on the association between *MIF* promoter variations and the risk of developing rheumatic heart disease (RHD) remain inconclusive. RHD is an important complication of streptococcal infections in the Middle East, not least in Saudi Arabia, and identifying risk markers is an important priority. Therefore, we investigated the association between two functional *MIF* promoter variations and RHD susceptibility and severity in Saudi patients: the *MIF*-173G > C substitution (rs755622) and the *MIF*-794 CATT_5–8_ tetranucleotide repeat (rs5844572). Three hundred twenty-six individuals (124 RHD patients and 202 age-, sex-, and ethnically matched healthy controls) were genotyped using allelic discrimination and fragment analysis. Data were analyzed with respect to disease susceptibility, severity, sex, and age of onset. There was a significantly lower frequency of 173C allele carriage in RHD patients compared to controls [odds ratio (OR) = 0.47; 95% confidence intervals (CIs) = 0.28–0.77; *p* = 0.003]. Interestingly, the 173C allele was associated with late disease onset (*p* = 0.001). The 794 5-repeat allele was associated with decreased RHD risk (OR = 0.56; 95% CIs = 0.38–0.82; *p* = 0.003). In contrast, the 794 6-repeat allele was associated with increased risk of RHD (OR = 1.7; 95% CIs = 1.2–2.5; *p* = 0.002). *MIF* promoter variations appear to have a dual role in RHD, with 173C allele non-carriers at higher risk of developing RHD at a younger age. These results require further validation in larger multi-ethnic cohorts, and functional studies are necessary to understand the underlying molecular mechanisms driving the at-risk phenotype.

## Introduction

Rheumatic heart disease (RHD) is an autoimmune disease that can develop following throat infections with one of the group A beta-hemolytic streptococci (GAS). Although standard antibiotic treatments are effective and help to prevent the disease, RHD is a common source of acquired pediatric heart disease in many developing nations (1), to the extent that the World Health Organization (WHO) (2) and World Heart Federation (WHF) (3) recognize RHD as a neglected tropical disease (NTD). It is estimated that GAS infection is a major global human pathogen that causes morbidity and mortality only exceeded by HIV, TB, and malaria (4). RHD is also an important and ongoing cause of cardiac disease in indigenous populations in developed countries (5, 6). For example, in a hospital-based study of children admitted with rheumatic fever (RF), the precursor of RHD, in Saudi Arabia, carditis was reported in 53% and severe carditis in 32% of cases (7). RF was more common in children from urban, low-income, and densely populated areas. In another study in western Saudi Arabia, Al-Sekait et al. assessed the prevalence of RHD in 9418 school children aged between 6 and 15 years (8) (representing 10% of the target population) and showed that RHD prevalence was 2.4/1000 (8). Molecular mimicry is thought to trigger RHD (9); however, the exact mechanisms underlying autoimmunity and cardiac tissue damage remain unknown.

Of note, only some GAS strains are rheumatogenic (10), and only 50% of individuals infected with these strains develop RHD (9). Furthermore, the disease appears to have a familial component: familial predisposition in RF was reported as early as 1889 by Cheadle, who estimated a five times greater risk of developing the disease in individuals with a family history compared to those without a family history (11). A meta-analysis of twin studies estimated that 60% of RF risk was heritable (12), which is higher than some other well-characterized complex autoimmune diseases (13). These data highlight the importance of a genetic component to the disease, and genetic variability in inflammatory and immune genes has been associated with RHD development and severity (10, 14–16).

Macrophage migration inhibitory factor (MIF) is a pleiotropic immune mediator important in innate and adaptive immune responses. *MIF* is a small gene (<0.7 kb) of three exons located on 22q11.2, and it is highly conserved across different species (17). MIF expression is rapidly induced by low concentrations of Gram-positive staphylococcal and streptococcal components (18), resulting in the production of a variety of cells types including macrophages, lymphocytes, and epithelial cells. MIF exerts a wide range of proinflammatory activities by upregulating expression of a variety of immune mediators including TLR4, IL-6, and TNFα (17, 19) and overriding the immunosuppressive activity of glucocorticoids (17). Studies on infarcted myocardium have shown opposing actions for MIF depending on disease stage and its cellular source (20). For example, MIF is cardioprotective when cardiac ischemia/reperfusion is brief (21), but when ischemia is prolonged, MIF activates immune cells and increases inflammation (22).

The *MIF* promoter contains two functional variations that affect its gene expression and protein levels *in vitro* and *in vivo*: a *MIF*-173G > C substitution (rs755622) and a *MIF*-794 CATT_5–8_ tetranucleotide repeat (rs5844572) (23, 24). The C allele at the 173 position is associated with higher *MIF* expression in T cell lines; however, in a lung carcinoma cell line, the G allele was associated with higher gene expression (25). *In vitro* and *in vivo* studies have shown that a greater number of CATT repeats are associated with higher gene expression (17, 24).

Migration inhibitory factor is pathogenic in several diseases including infectious (26), autoimmune (17), and cardiovascular diseases (20). In infectious diseases, MIF promotes pneumococcal clearance (26); however, MIF alleles that promote gene expression are associated with severe malarial anemia (27). MIF plays a dual role in autoimmune diseases; for example, alleles that promote gene expression protect against systemic lupus erythematosus (SLE) but, in patients with established disease, alleles that reduce expression protect against disease complications and multiple organ involvement (28). Studies on the genetic factors that contribute to RHD risk and severity are scarce, especially in the developed world. Therefore, we tested the hypothesis that *MIF* promoter variations modulate the course of RHD by evaluating these sequence variants in relation to RHD susceptibility, severity, and age of onset in 326 Saudi Arabian participants.

## Materials and Methods

### Study Population

One hundred and twenty-four RHD patients were enrolled in a pilot study at the Pediatric Cardiology Clinic at the Maternity and Children Hospital, Al-Madinah region, Saudi Arabia between March 2013 and June 2014. The Maternity and Children Hospital ethics committee approved the study protocol, and the authors followed the norms of the World Medical Association Declaration of Helsinki. All adult patients and donors or the parents or guardians of child participants (<18 years old) signed a fully informed and written consent form approved by the ethics committee.

The patient and control cohorts are described in previous studies (15, 16). Briefly, diagnosis was made according to the modified Jones criteria at initial diagnosis and confirmed by echocardiography (29). Patients were subgrouped according to echocardiographic findings into either mitral valve lesion (MVL) or multiple valve lesions including the mitral valve, termed a “combined valve lesion” (CVL). Exclusion criteria were patients with RF but without valvular disease, heart complications, or other inflammatory conditions. Age of onset was the age at which the patient had his/her first diagnosis confirmed by echocardiography. The control group was a random population sample of 202 healthy volunteers identified *via* the National Blood Service in the Al-Madinah region. Controls were age-, gender-, and ethnically matched unrelated volunteers with no history of cardiac or autoimmune diseases. All participants were of Saudi Arabian ethnicity.

### Genotyping MIF Promoter Polymorphisms

DNA was purified from 2-ml whole blood using the QIAamp DNA Mini Kit (Qiagen, Hilden, Germany) according to the manufacturer’s protocol. Extracted DNA was quantified by spectrophotometry (MaestroNano, MaestroGen, Las Vegas, NV, USA) and stored at −20°C until use. The *MIF*-173 polymorphism was genotyped using the duplex quantitative TaqMan 5′ Allelic Discrimination Assay (Applied Biosystems, Foster City, CA, USA; assay ID C_2213785_10) as directed by the manufacturer. Briefly, assays were performed in a final volume of 10 μl (including TaqMan Genotyping Master Mix, 40× SNP Genotyping Assay Mix, DNase-free water, and 20-ng DNA) in 96-well plates using the following amplification protocol: 95°C for 10 min followed by 50 cycles at 95°C for 15 s and 60°C for 1 min (annealing/extension). Fluorescence detection took place at 60°C. Non-template controls were included in each run. The genotype call rate was over 99%. Duplicate genotyping of 10% of samples selected at random was performed for quality control. Assays were performed using the StepOnePlus system, and the automated Sequence Detection Software (SDS) v2.3 was used for auto-calling (Applied Biosystems).

The *MIF*-794 repeat was genotyped by conventional PCR, and the product was separated by capillary electrophoresis (3500 Genetic Analyzer, Life Technologies, Carlsbad, CA, USA). Reactions were performed with 10 pmol of each primer: forward primer (FAM-5′-AAATCTCTGAGGACCTGGCC-3′) and reverse primer (5′-CACCGTGTATGGCCTCTCAT-3′) designed using Primer3.^1^ PCR products were detected by capillary electrophoresis using the fluorescently labeled forward primer. Amplifications were carried out in a 10-μl reaction volume containing 30-ng genomic DNA in 2× Go-Taq Master Mix including MgCl_2_, 10× PCR buffer, dNTPs, and 10 units of Taq DNA polymerase (M7132, Promega, Madison, WI, USA). Samples were amplified using the Veriti thermal cycler (Life Technologies, Carlsbad, CA, USA). The cycling and amplification conditions were initial denaturation at 95°C for 2 min followed by 35 cycles with denaturation at 95°C for 15 s, annealing at 60°C for 15 s, and extension at 72°C for 30 s. Automated capillary electrophoresis on the 3500 Genetic Analyzer was performed for each sample following the manufacturer’s protocol, and the CATT alleles were identified using GeneMapper v4.1 software (Life Technologies).

### Statistical Analysis

Statistical analyses were conducted using SPSS version 17 (IBM Statistics, Chicago, IL, USA). In addition, results were confirmed using a freely available online statistical tool, VassarStats.^2^ Genotyping data were checked for any deviation from Hardy–Weinberg equilibrium (HWE) using chi-squared contingency tables. Genotype and allele frequencies were determined by direct counting in patients and controls. Significant differences in the distribution of *MIF* promoter polymorphisms between cases and controls were tested using chi-squared contingency tables or Fisher’s exact test as appropriate. Linear regression was used to test for any association between the genetic variations and age of onset (the dependent variable was age of onset and allele frequencies were predictors). Odds ratios (ORs) and 95% confidence intervals (CIs) were calculated. A *p*-value <0.05 was considered statistically significant.

## Results

### Patient Characteristics

The demographic and baseline characteristics of the patients at disease presentation according to echocardiographic findings are presented in Table 1. Three hundred twenty-six Saudi Arabian individuals (202 controls and 124 patients) were genotyped for the *MIF*-173 (rs755622) and *MIF*-794 (rs5844572) polymorphisms. RHD patients were 56% males and 44% females, and 67 patients (54%) had MVL and 57 patients (46%) had CVL. Carditis (64%) and arthritis (57%) were present in the majority of patients. Acute phase reactants were elevated in 79% of patients at presentation (Table 1).

**Table 1:** **Demographic characteristics and clinical details of the patients (*n* = 124) and controls (*n* = 202)**.

Parameter	Value
**Average age (mean ± SD years)**	
Controls	20.5 ± 4.2
Patients (at follow-up)	19 ± 5
Patients (age of onset at diagnosis)	7.9 ± 2.4
**Gender: male/female (%)**	
Controls	55/45
Patients	56/44

**Clinical manifestations**	***N* cases (%)**

**Characteristics**	
Valvular lesion	
Mitral valve lesion (MVL)	67 (54)
Combined valve lesion (CVL)	57 (46)
Carditis	79 (64)
Arthritis	71 (57)
Chorea	14 (11)
Skin rash	3 (2)
Subcutaneous nodules	2 (2)
Recurrence	NA
**Laboratory findings at presentation**	
Elevated acute phase reactants (CRP/ESR)	98 (79)
Prolonged PR interval	68 (55)

**N*, number; *N* cases, number of cases; NA, not available; CRP, C-reactive protein; ESR, erythromycin sedimentation rate*.

### Distribution of MIF Polymorphisms

The genotypes, allele frequencies, and allele carriage distributions are shown in Table 2. There were no deviations from the HWE in patients or controls for either *MIF* variant. The genotype and haplotype frequencies of *MIF* promoter polymorphisms in the control group were consistent with similar published studies from other countries; however, this is the first report of frequencies of these polymorphisms in a Saudi Arabian or Arab population. The allelic frequencies of the 173 polymorphism in healthy controls were similar to those reported in studies from Turkey, the Netherlands, and Caucasians from New Zealand (30–32), but different to those reported in a Mexican population (33). Similarly, distributions of the 5-, 6-, 7-, and 8-CATT alleles located at the 794 locus were comparable to those reported from Italy and Belgium (34), but different to Mexican and Japanese populations (33, 35).

**Table 2:** **Distribution of *MIF*-173 polymorphism genotypes and allele frequencies in patients and controls**.

*MIF*-173	Control (*N* = 202)		Patients (*N* = 124)		χ^2^	df	*p*-value	OR (95% CI)
	Count	Frequency	Count	Frequency				
**Genotype**								
GG	122	0.60	95	0.77	10.2	2	**0.006**	
GC	64	0.32	26	0.21				
CC	16	0.08	3	0.02				
**Allele frequency**								
G	308	0.76	216	0.87	Ref.			
C	96	0.24	32	0.13	11.5	1	**0.0007**	0.48 (0.31–0.74)
**Allele carriage**								
							
(GG + GC) vs. CC	186	0.92	121	0.98	4.2	1	0.04	3.5 (0.99–12.2)
(CC + GC) vs. GG	80	0.4	29	0.23	9.1	1	**0.003**	0.47 (0.28–0.77)

*Significant findings are shown in bold; *N*, number*.

### Relationship between *MIF* Polymorphisms and RHD

The *MIF* polymorphism genotype and allele frequencies were different in patients with RHD. There was a significant difference in the distribution of the *MIF*-173 genotype frequency between patients and controls (χ^2^ = 10.2, *p* = 0.006; Table 2). *MIF*-173C allele frequencies were associated with a decrease in RHD risk (χ^2^ = 11.5, *p* = 0.0007, OR = 0.48, 95% CIs 0.31–0.74). Likewise, 173C allele carriage (CC + GC vs. GG) was associated with a decreased risk of developing RHD (χ^2^ = 9.1, *p* = 0.003, OR = 0.47, 95% CIs 0.28–0.77).

The genotypic and allelic frequencies for the *MIF*-794 CATT_5–8_ repeat variation in RHD and healthy subjects are shown in Table 3. On the one hand, the 794CATT_5_-allelic frequencies were significantly higher in the control group than RHD patients (χ^2^ = 8.8, *p* = 0.003, OR = 0.56, 95% CIs 0.38–0.82). On the other hand, 794 CATT_6_-allelic frequencies were associated with an increased risk of developing RHD (χ^2^ = 9.7, *p* = 0.002, OR = 1.7, 95% CIs 1.2–2.5). Most evidence indicates that 794 CATT_6–8_ repeats have higher transcriptional activity than 794 CATT_5_ repeats; therefore, we grouped 794 CATT_6–8_ repeats and termed them the X allele. X-allele frequency was more common in RHD subjects than in healthy controls (χ^2^ = 8.8, *p* = 0.003, OR = 1.8, 95% CIs 1.22–2.66).

**Table 3:** **Distribution of *MIF*-794 repeat genotypes and allele frequencies in patients and controls**.

*MIF*-794[CATT]	Control (*N* = 202)		Patients (*N* = 124)		χ^2^	*p*-value	OR (95% CIs)
	Count	Frequency	Count	Frequency		
**Genotype**								
5/5	19	0.09	3	0.02	5.96	**0.02**	0.24 (0.07–0.82)
5/6	64	0.32	35	0.28	0.4	0.5	0.9 (0.5–1.4)
5/7	7	0.03	3	0.02	0.3	0.6	0.7 (0.18–2.7)
5/8	4	0.02	0	0.00	2.5	0.1	0.98 (0.96–1)
6/6	82	0.41	69	0.56	7.0	**0.008**	1.8 (1.17–2.9)
6/7	18	0.09	10	0.08	0.07	0.8	0.9 (0.4–2)
6/8	6	0.03	1	0.01	–	0.3	–
7/7	2	0.01	2	0.02	–	0.6	–
7/8	0	0.00	0	0.00	–	–	–
8/8	0	0.00	1	0.01	–	–	–
**X allele carriage**								
(5/5 + 5/X) vs. X/X	94	0.47	41	0.33	5.7	**0.02**	0.57 (0.36–0.9)
(X/X + 5/X) vs. 5/5	183	0.91	121	0.98	6.0	**0.01**	4.2 (1.2–14.5)
**Allele freq.**								
5	113	0.28	44	0.18	8.8	**0.003**	0.56 (0.38–0.82)
6	252	0.62	184	0.74	9.7	**0.002**	1.7 (1.2–2.5)
7	29	0.07	17	0.07	0.03	0.9	1 (0.51–1.8)
8	10	0.02	3	0.01	–	0.4	–
X allele freq.	291	0.82	204	0.72	8.8	**0.003**	1.8 (1.22–2.66)

*Significant findings are shown in bold; *N*, number; X refers to 794 CATT alleles 6–8*.

### MIF Genotype Distribution and Age of Disease Onset

**Figure 1:**
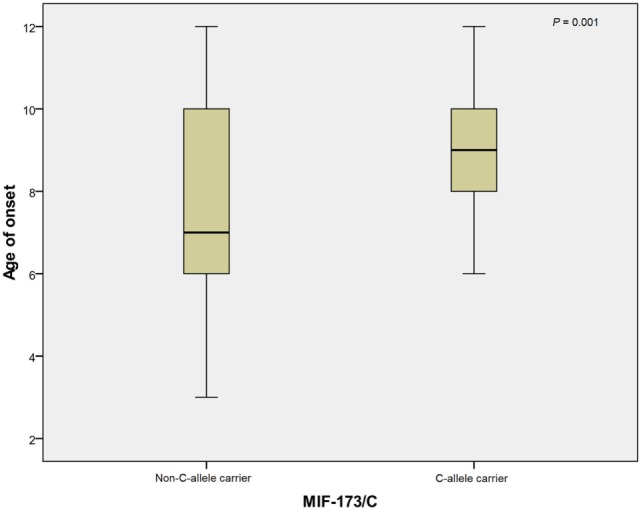
**Mean of age at onset for 173C allele non-carriers (*N* = 94) and carriers (*N* = 28)**. The mean age at onset of non-C allele carriers and C allele carriers were 7.5 ± 2.4 and 9.2 ± 1.6, respectively. *N*, number.

In linear regression analysis, the age of onset was significantly greater for *MIF*-173C allele carriers than non-*MIF*-173C allele carriers in RHD patients (*p* = 0.001; Figure 1). The mean ages of onset of C-allele carriers and non-carriers were 9.2 ± 1.6 and 7.5 ± 2.4, respectively. There was no significant association between the tetranucleotide variation and disease age of onset (data not shown).

## Discussion

Accumulation of deleterious and disease-associated genetic variations in immune genes has been linked to autoimmune disease susceptibility (36). RHD is a multifactorial disorder involving multiple genetic and environmental factors. Although it remains a common autoimmune heart disease in children in developing countries, only a few studies from these regions have explored the influence of genetic variations in immune genes in RHD (37).

In this pilot case–control study, the impact of *MIF* functional variations was evaluated with respect to RHD susceptibility and severity in 326 Saudi Arabian individuals (202 controls and 124 patients). We report that the pleiotropic inflammatory cytokine MIF exerts dual effects in RHD patients. The 173C allele was associated with reduced RHD risk and, interestingly, this genotype was associated with later disease onset. In contrast, the 794 CATT_6_ allele was associated with increased RHD risk, but the 794 CATT_5_ allele was associated with reduced RHD risk. Genetic variability in *MIF* may have a dual impact on the development of RHD in the Saudi Arabian population.

The pleiotropic roles of MIF and *MIF* alleles on the immunopathogenesis of infectious and autoimmune diseases have been documented. For example, in SLE, MIF alleles associated with higher gene expression (173C and 794 extended alleles) are associated with a lower risk of SLE (38). However, in patients with established disease, lower expression alleles are associated with reduced end-stage organ involvement (28). Interestingly, the *MIF*-173C allele has been associated with a lower incidence of antinuclear antibody (ANA) development in SLE, supporting a protective role for MIF in SLE (17). On the other hand, a meta-analysis has shown that 173 polymorphisms are a risk factor for inflammatory bowel disease; however, due to a lack of clinical details and original data, the effect of *MIF* polymorphisms on disease progression and severity could not be analyzed (39). Similarly, MIF-794 allele 7 has been shown to be a risk factor for the development of rheumatoid arthritis; however, 794 allele 5 is correlated to reduced disease severity (33, 40). In another autoimmune disease, systemic sclerosis, the *MIF*-173 polymorphism was associated with the development of the diffuse and more severe form of the disease in a large meta-analysis (41). The role of MIF in different diseases is clearly highly cell- and disease-context dependent.

*MIF* polymorphisms and protein expression also have an impact on outcomes in patients with infectious diseases. In African and Indian studies, longer CATT repeats were associated with malarial complications, such as severe malarial anemia (27) and cerebral malaria (42), which are the main causes of death in these patients. Interestingly, the frequency of the shortest CATT-5 repeat was greatest in African populations, leading to the speculation that MIF low expression alleles may be protective against malaria, similar to the effect seen with sickle hemoglobin (HbS) genes (17).

To our knowledge, this is the first case–control study of the impact of *MIF* promoter variations on RHD risk. One Turkish study explored the influence of the 173 polymorphism on acute RF, the precursor of RHD, and reported that the polymorphism conferred increased risk (43). However, the authors did not report any clinical details on RHD in this cohort. Unfortunately, we could not examine the effect of *MIF* on RF since our patients were recruited from a tertiary cardiac center that only receives patients with established valvular disease. Nevertheless, it has been shown that MIF plays an important role in heart disease (44). In myocardial infarction (MI), myocardiocyte-secreted MIF exerts a protective effect when cardiac ischemia is brief by activating AMP-activated protein kinase (AMPK) and stress suppression (44). AMPK phosphorylation promotes glucose uptake *via* glucose transporter-4 (GLUT4), thereby providing metabolic protection (45). However, in prolonged ischemia, MIF activates immune cells and increases inflammation, cardiac remodeling, and fibrosis by promoting matrix protein synthesis by myofibroblasts (45). In RHD, valvular damage is caused by recurrent rheumatic insults. In some patients, persistent inflammation leads to the formation of Aschoff bodies, which are granulomatous lesions characterized by mononuclear cell and macrophage infiltrates in the myocardium and endocardium. Over the disease course, the valves become thickened and, eventually, stenosed. MIF’s role in these inflammatory foci has yet to be studied, but it is possible that, in the early stages of the disease, MIF might help to clear certain pathogens and apoptotic cells (46). In turn, this could help to protect cardiomyocytes and delay valvular damage. Conversely, recurrent rheumatic insults in the presence of MIF may increase inflammatory cell recruitment and promote proinflammatory mediators, thereby enhancing regional inflammation and damaging cardiac tissue.

Of particular note, the *MIF*-173C allele was associated with later disease onset as well as decreased risk of RHD. To our knowledge, this is the first study to examine the effect of polymorphisms on the age of onset of RHD. These data suggest that MIF action may be age dependent; however, the results must be considered with caution since there are different definitions of “age of onset,” and advanced diagnostic echocardiography has influenced RHD burden data (47, 48). Several studies have shown that MIF is age dependent in other diseases: adults with a low-expressing MIF allele (CATT5) are at high risk of Gram-negative bacterial infections (49) and high-expressing MIF genotypes protect older patients from sepsis mortality (50). In animal models, the protective role of MIF during ischemic injury is abolished in senescent hearts, an effect correlated to impaired AMPK activation and low MIF expression levels (51). Interestingly, exogenous MIF can effectively rejuvenate stem cells isolated from age-induced senescent animals (52). Clearly, MIF variations influence disease severity and progression; therefore, future clinical studies need to collect and analyze data that includes disease stage and patient age.

Here, we report an association between *MIF* promoter variations and RHD. These findings require replication in larger, multi-ethnic studies to overcome some of the ethnic bias in our study, the retrospective analysis, and to provide sufficient power for robust subgroup analysis. Furthermore, we only analyzed two polymorphisms and not the entire *MIF* gene, and it would be useful to analyze other *MIF* polymorphisms and functional genetic variations in downstream signaling pathways to fully elucidate the function of this pathway in RHD. Finally, studies from Arab countries are scarce with respect to *MIF* variations in any infectious or autoimmune diseases, making ethnic comparisons difficult and only permitting comparisons with results from non-Arab populations.

In conclusion, *MIF* promoter variants may have an impact on RHD susceptibility, severity, and age of onset. Further prospective, multi-ethnic studies are required.

## Author Contributions

AA designed the study, planned and performed experiments, analyzed data, and wrote the manuscript. NA-H and AA-M collected patient samples and clinical data and contributed to research discussion. AE and WA-H analyzed data and edited the manuscript. YA and KA-H designed the study, contributed to research discussion, and edited the manuscript.

## Conflict of Interest Statement

The authors declare that the research was conducted in the absence of any commercial or financial relationships that could be construed as a potential conflict of interest.

## Footnotes

^1^http://primer3.ut.ee/

^2^www.vassarstats.net
